# Burst initiation and propagation in cortical cultures requires an inhomogeneous connectivity distribution and synaptic rescaling

**DOI:** 10.1186/1471-2202-12-S1-P85

**Published:** 2011-07-18

**Authors:** Sarah J Jarvis, Stefan Rotter, Ulrich Egert

**Affiliations:** 1 Bernstein Center Freiburg, Freiburg 79104, Germany; 2 Biomicrotechnology, Department of Microsystems Engineering - IMTEK, University of Freiburg, 79110 Freiburg, Germany; 3 Computational Neuroscience, Faculty of Biology, University of Freiburg, 79104, Germany

## 

Dissociated cortical cultures grown on microelectrode arrays (MEA) have been established as a useful biological model in the analysis of network dynamics. Their network dynamics are dominated by periods of strongly synchronized spiking, termed 'bursting', whose role is currently not understood. It has been demonstrated that bursts have different motifs and contain structure, refuting the possibility that they are merely chaotic activity. Of particular interest are the conditions required for bursting to initiate and propagate throughout the entire network. Within cultures, initiation sites can be well localized, even under varying experimental conditions such as cell density and network size. Propagation waves display fairly regular patterns of neuron recruitment within the network burst. However, in order to minimize bursting and promote closed-loop communication with the disassociated culture, it is of interest to understand what conditions are necessary for bursting to arise.

Interestingly, the propagation of bursts has been observed to be faster than can be accounted for by only local connectivity. While paired intracellular recordings have revealed some clues as to the local structure and short range connectivity, they were unable to clarify the contribution of long-range connectivity of neurons to burst propagation. Additionally, pharmacological studies which result in freezing of synaptic plasticity and impaired cell migration have demonstrated that modifications to connectivity can greatly alter the pattern of burst propagation.

As network structure has been established to strongly affect dynamics, we investigate the contribution of network features that can account for experimentally observed burst initiation and propagation patterns. Specifically, we examine the contribution of long-range connections within a 2D model network of spiking neurons representing a mature dissociated cortical culture. We previously demonstrated with networks of rate-based units that within clustered topologies, the relative number of long-range connection to cluster size greatly affects the robustness of the network to noise and ability to sustain activity [1], while including highly recurrent activity is required for fast ignition of activity from low-level background activity. Here, we extend these networks to a population of Integrate and Fire neurons and chart the effect of introducing inhomogeneities and a non-uniform distribution of connections, specifically the numbers and location of post-synaptic connections of each neuron. By driving the network with low levels of background activity, we observe the emergence of burst initiation sites and chart how different cluster configurations act to alter their location while simultaneously inspecting the velocity of burst propagation as it spreads throughout the network. Then, in keeping with recent evidence of synaptic reorganization in cortical cultures [2], we compensated the synaptic strength of neurons that received fewer presynaptic connections and noted how this increased the likelihood of inducing bursting. We compare burst profiles obtained in these simulations against their biological equivalents to identify the necessary topological features required.
